# Supplementary figures and images for: Genetic deletion of calcium-independent phospholipase A2γ protects mice from diabetic nephropathy
Source: PLoS One. 2024 Oct 31;19(10):e0311404. doi: 10.1371/journal.pone.0311404 (PMC11527321; doi:10.1371/journal.pone.0311404)

S1 Figure

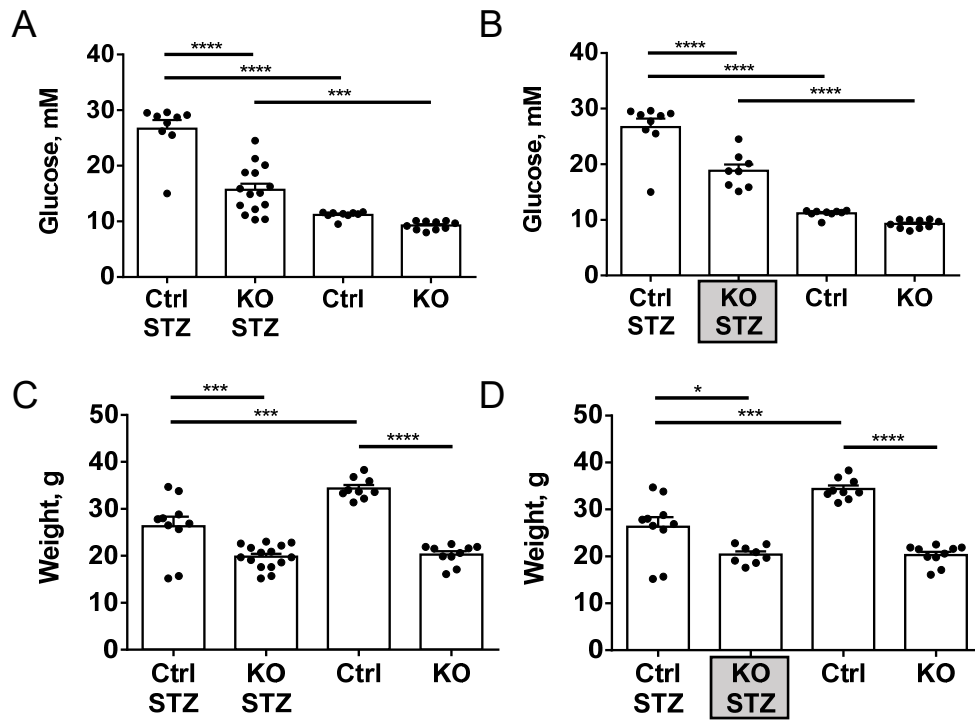

Supplement: S1 Fig — A) STZ induces significant increases in blood glucose in both control and iPLA2γ KO mice; glucose levels are higher in control mice. N = 9 mice in control (Ctrl) untreated (Untr), 10 in KO Untr, 10 in Ctrl STZ and 15 in KO STZ groups. B) Blood glucose levels after removal of the 7 STZ-treated KO mice with smaller increases in blood glucose levels (N = 8). C) Untreated and STZ-treated KO mice show lower body weights compared to untreated and STZ-treated controls. STZ-treated control mice show lower body weight compared to untreated control. D) Body weights after removal of the 7 STZ-treated mice with smaller increases in blood glucose. The glucose and body weights of each mouse represent mean values over the study period. *P<0.05, ***P<0.001, ****P<0.0001 (ANOVA). Since the blood glucose data in the Ctrl STZ and Ctrl groups was not normally distributed (panels A and B), we also used the Kruskal-Wallis test to confirm significant differences among groups (p<0.0001). (PDF) [file pone.0311404.s001.pdf]

S2 Figure

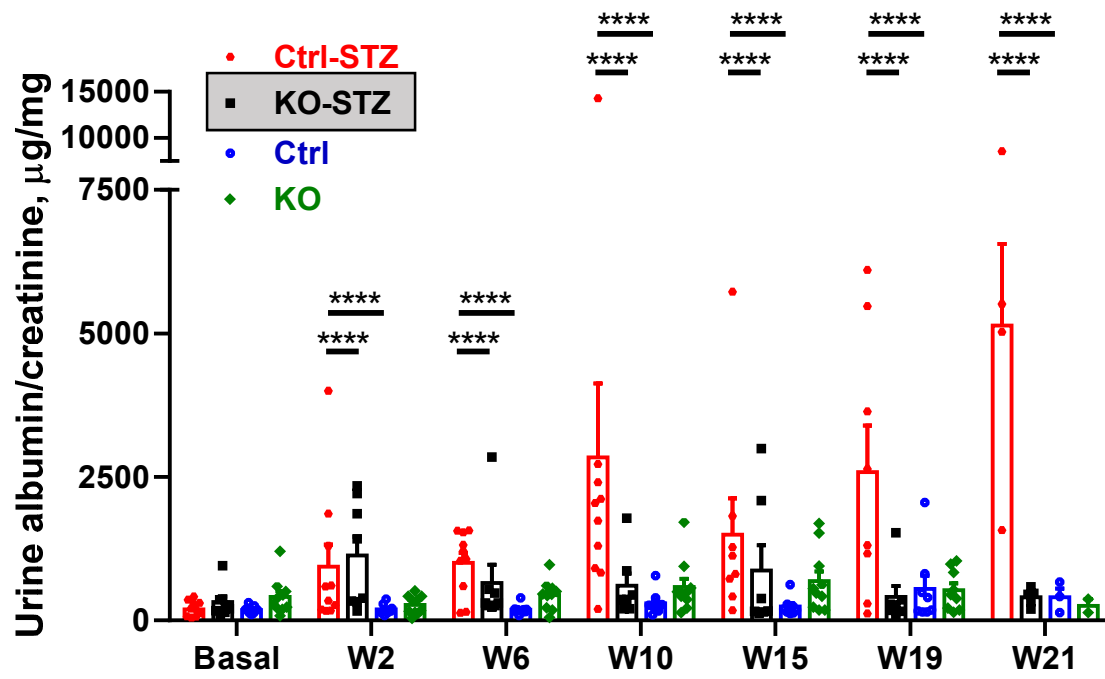

Supplement: S2 Fig — See legend to Fig 1. Results are presented after removal of 7 STZ-treated KO mice with smaller increases in blood glucose (N = 8 in KO STZ). The statistical significance of the changes remains the same as in Fig 1. (PDF) [file pone.0311404.s002.pdf]

S3 Figure

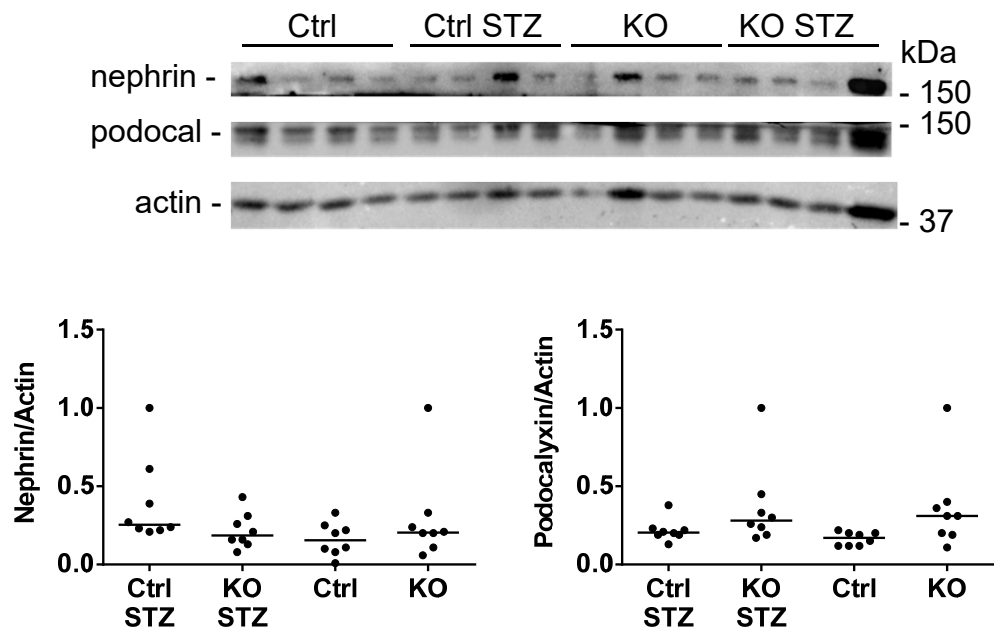

Supplement: S3 Fig — Glomerular lysates were immunoblotted with antibodies as indicated. Signals were quantified by densitometry. Bars indicate median values. There are no significant differences in nephrin and podocalyxin (Podocal) expression among groups (Kruskal-Wallis). There are 6–8 mice per group. (PDF) [file pone.0311404.s003.pdf]

S4 Figure

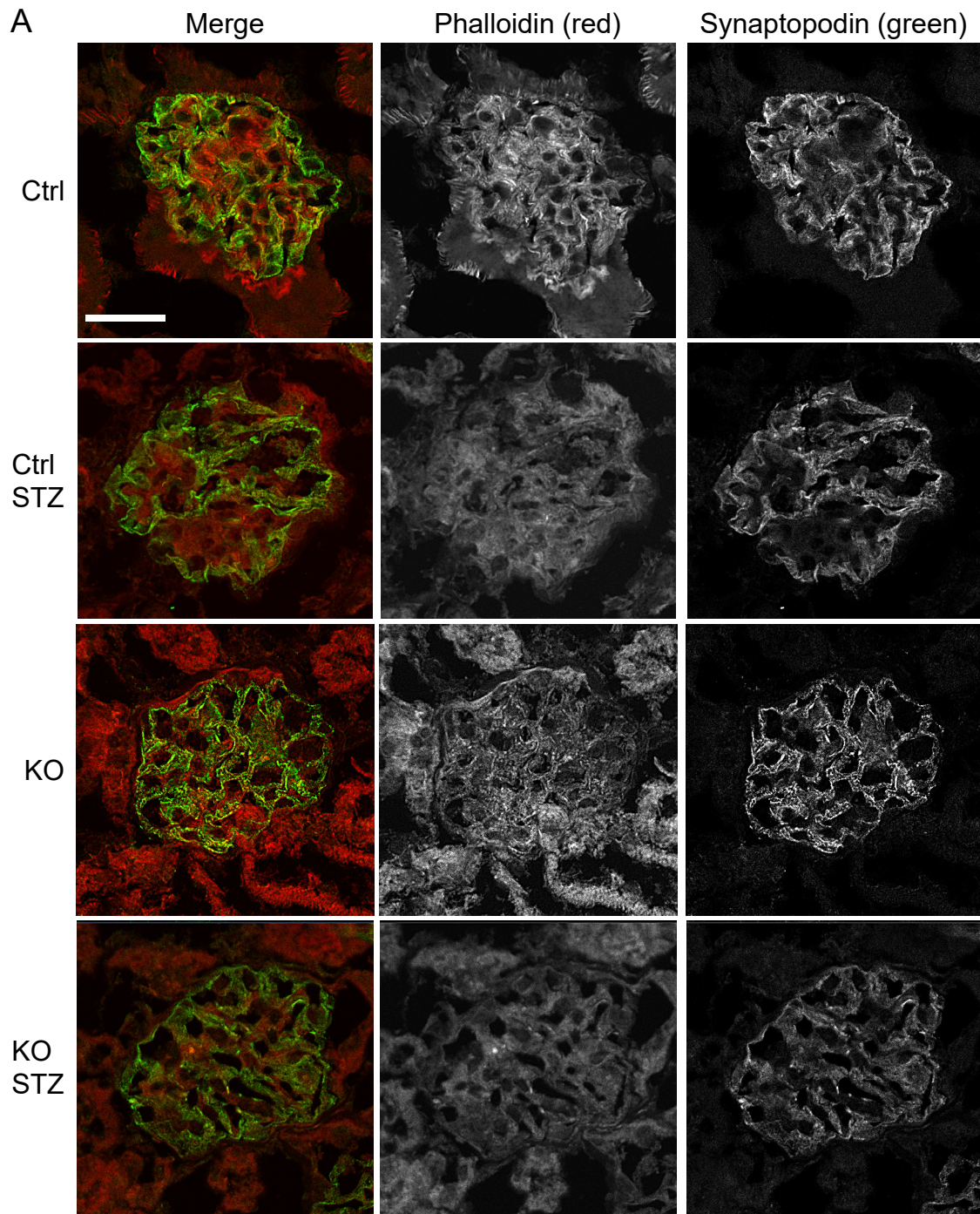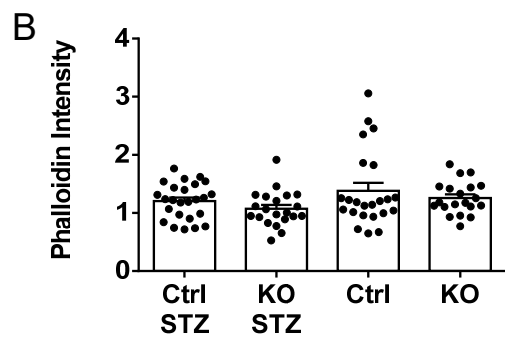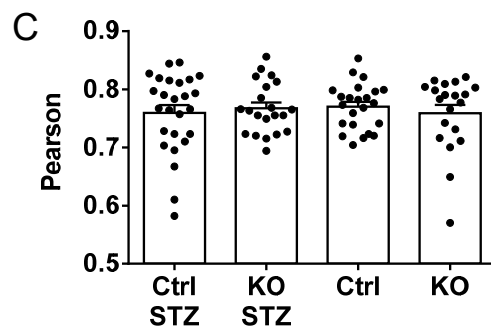

Supplement: S4 Fig — Kidney sections were stained with FITC-phalloidin, which reflects F-actin, and antibody to synaptopodin. A and B) Representative fluorescence micrographs and quantification of FITC-phalloidin fluorescence intensity are shown. Quantification of synaptopodin immunofluorescence intensity is similar to Fig 4. There are no significant differences in fluorescence intensity among groups. Pearson correlation coefficient for phalloidin and synaptopodin. There are no significant differences among groups (ANOVA or Kruskal-Wallis). 2–8 glomeruli/mouse in 4 mice per group were analyzed. Bar = 25 μm. (PDF) [file pone.0311404.s004.pdf]

S5 Figure

Control  
Untreated

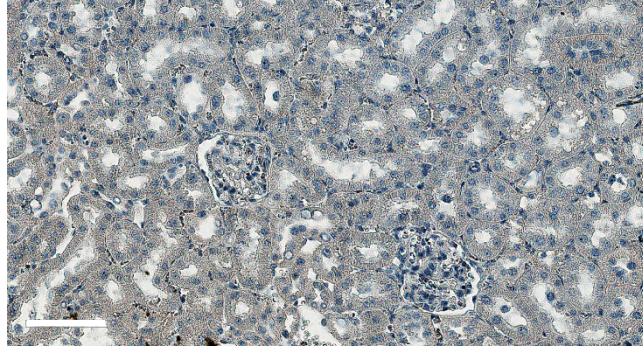

KO  
Untreated

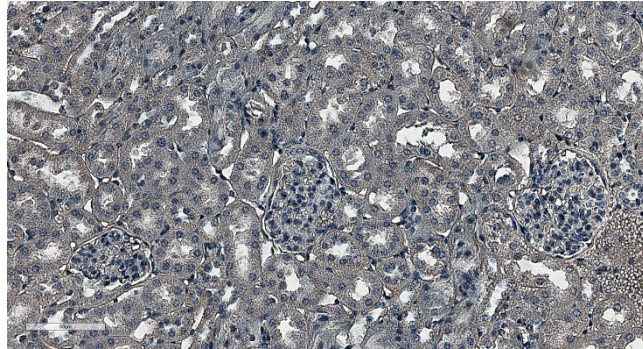

Control  
STZ

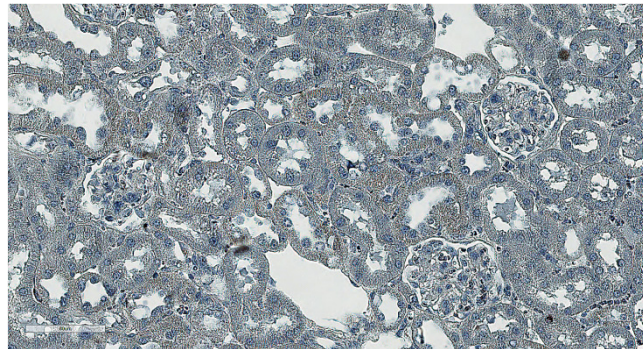

KO  
STZ

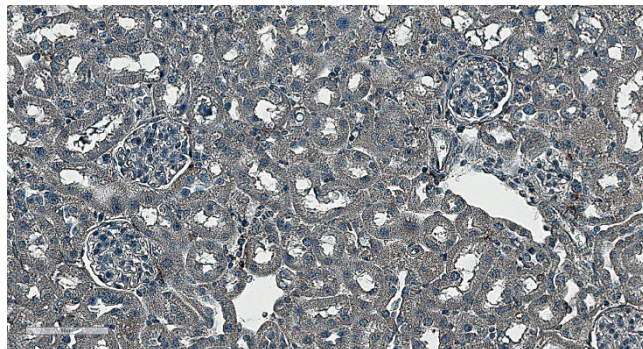

Spleen

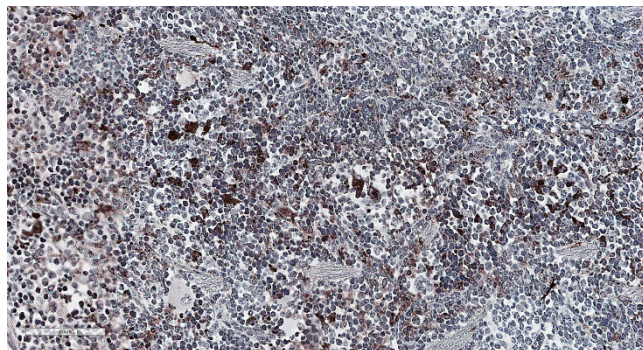

Supplement: S5 Fig — Kidney sections were stained with F4/80 antibody, which identifies macrophages. Representative micrographs are presented. There is only minimal F4/80 staining in the kidneys in the four groups of mice. Spleen is presented as positive control. Kidneys of 4–6 mice per group were examined. Bars = 80 μm. (PDF) [file pone.0311404.s005.pdf]

S6 Figure

Control STZ

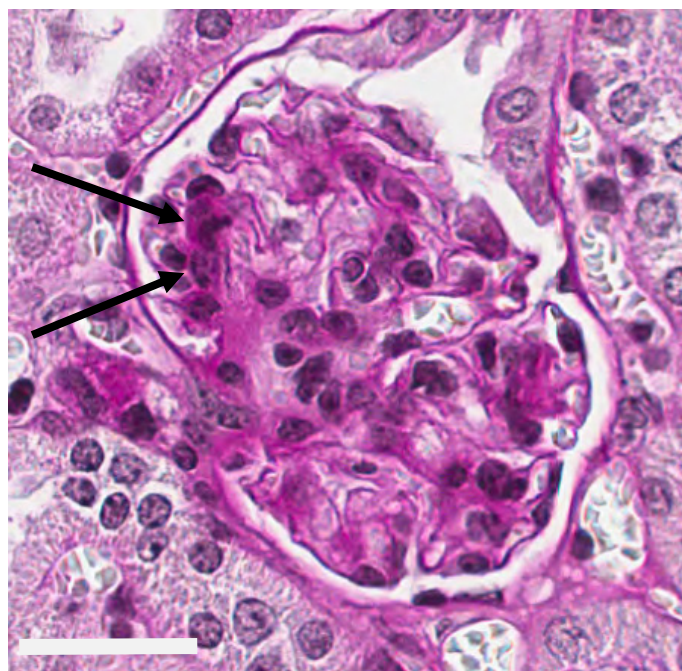

KO STZ

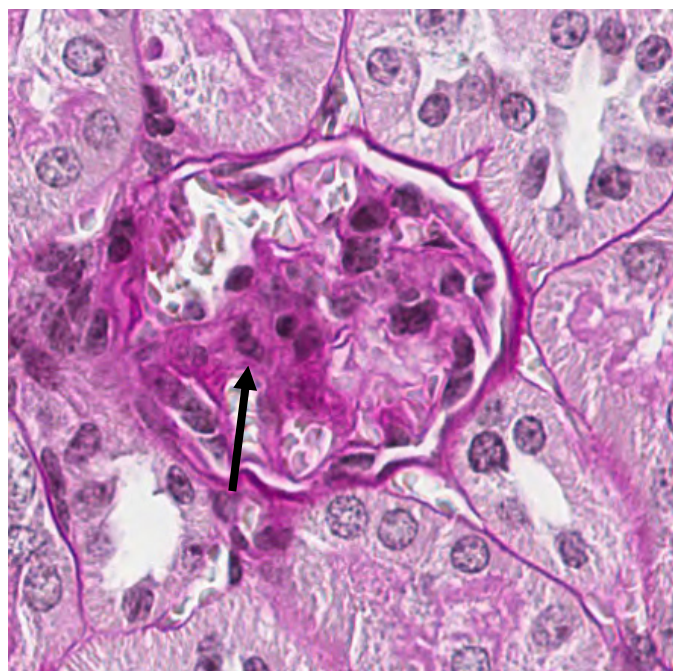

Supplement: S6 Fig — Kidney sections were stained with periodic acid-Schiff. Photomicrographs of diabetic control and KO mice showing neutrophils are presented. The neutrophils are labelled with arrows. Kidneys of 5–6 mice per group were examined. Bar = 30 μm. (PDF) [file pone.0311404.s006.pdf]

S7 Figure

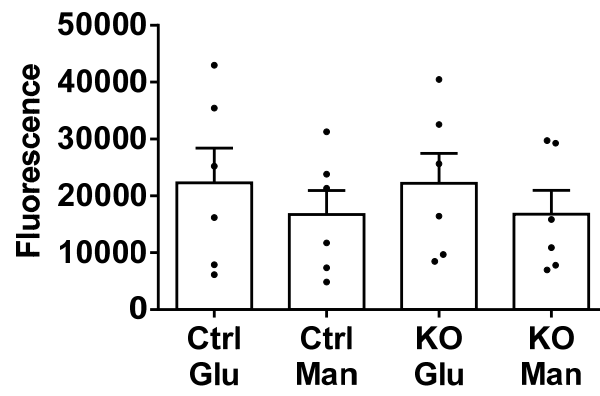

Supplement: S7 Fig — Total reactive oxygen species in control and iPLA2γ KO GECs were monitored by 2’,7’-dichlorodihydrofluorescein diacetate (DCF) staining. GECs were cultured in media containing high (36 mM) or low glucose (7.8 mM) plus mannitol for 24 h. DCF was added and fluorescence was measured after 15 min. There are no significant differences in DCF fluorescence between high and low glucose groups, nor between control and iPLA2γ KO GECs (ANOVA). 6 experiments. (PDF) [file pone.0311404.s007.pdf]

S8 Figure

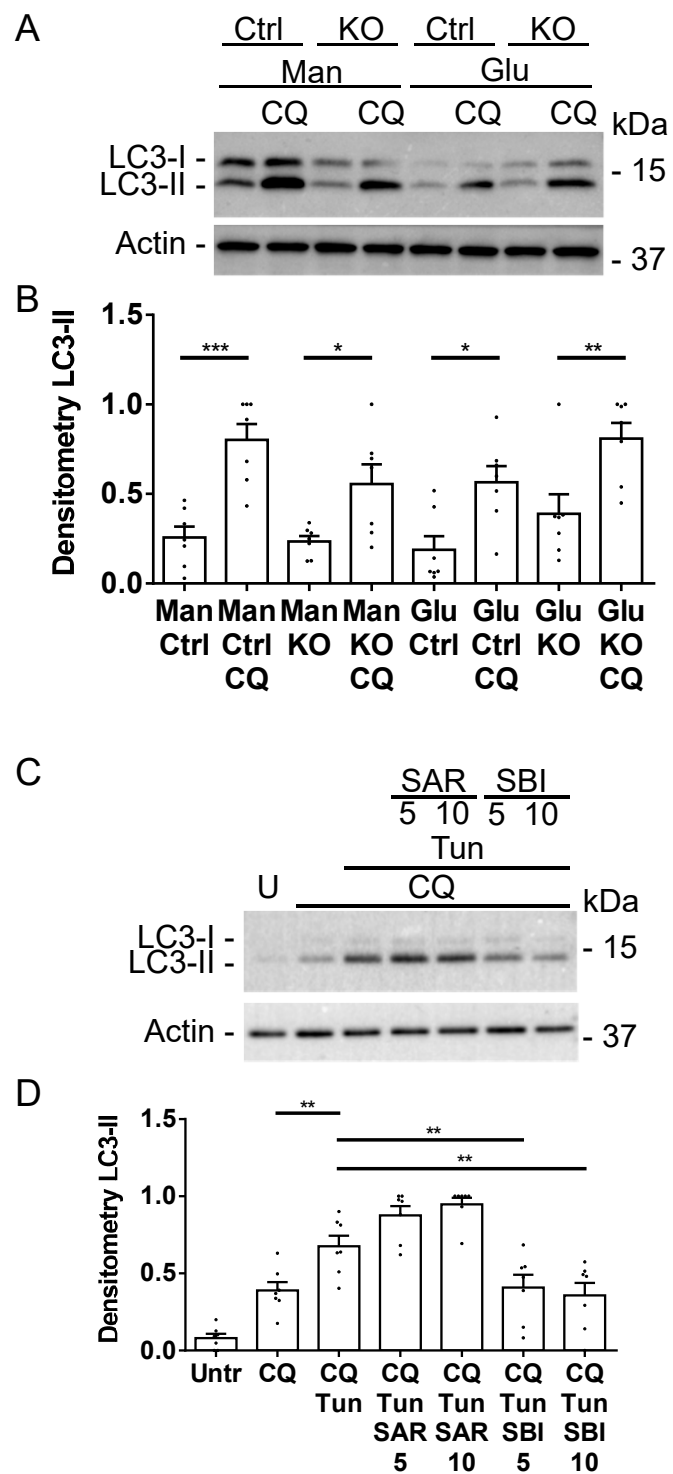

Supplement: S8 Fig — A and B) Control and iPLA2γ KO GECs were cultured in medium containing 7.8 mM glucose (Glu). Then, medium was switched to 7.8 mM glucose + 28 mM mannitol (Man) or high glucose (36 mM), and cells were treated with or without chloroquine (CQ; 25 μM) for 24 h. Cell lysates were immunoblotted with anti-LC3 antibody and signals were quantified by densitometry. LC3-II increases significantly after addition of CQ in control and iPLA2γ KO cells exposed to mannitol or high glucose; however, there are no significant differences among the 4 CQ-treated groups. *P<0.05, **P<0.01, ***P<0.001 (ANOVA). 4 experiments performed in duplicate (ANOVA). C and D) Control GECs were untreated (U) or were incubated with CQ (25 μM), tunicamycin (Tun, 1 μg/ml), Tun + SAR405 (SAR, 5 or 10 μM), or Tun + SBI0206965 (SBI, 5 or 10 μM) for 24 h. Cell lysates were immunoblotted as above. Tun increases LC3-II and the increase is blocked by SBI0206965, but not SAR405. **P<0.01 (ANOVA). 4 experiments performed in duplicate. (PDF) [file pone.0311404.s008.pdf]

Uncropped immunoblots

Figure 5

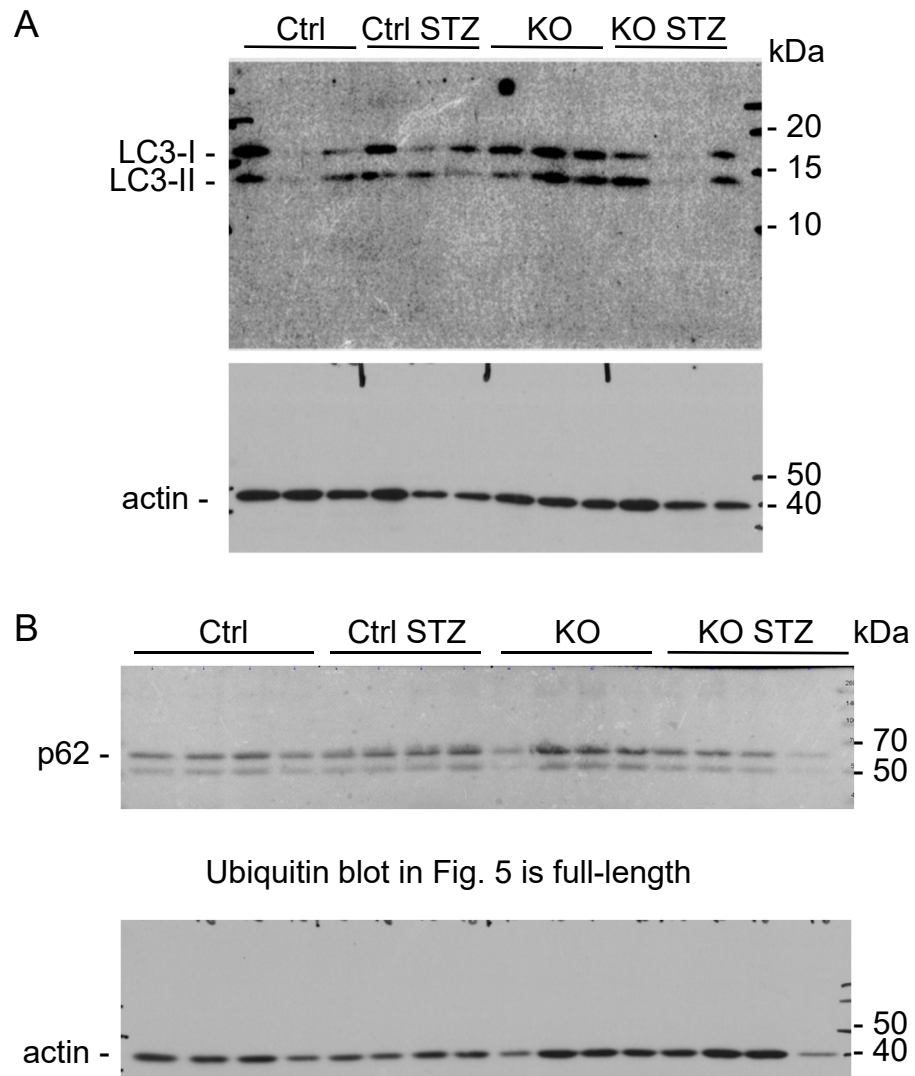

S3 Figure

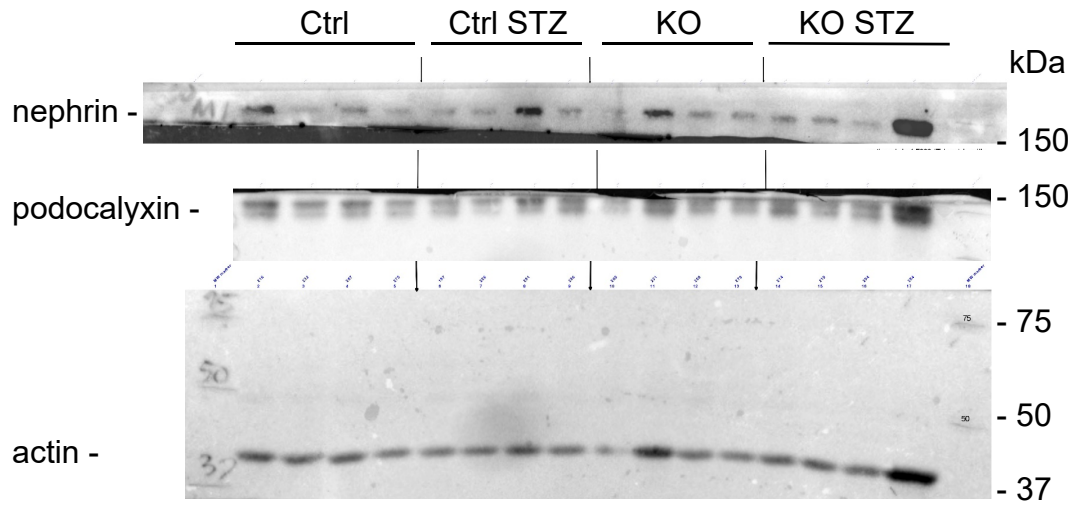

S8A and C Figures

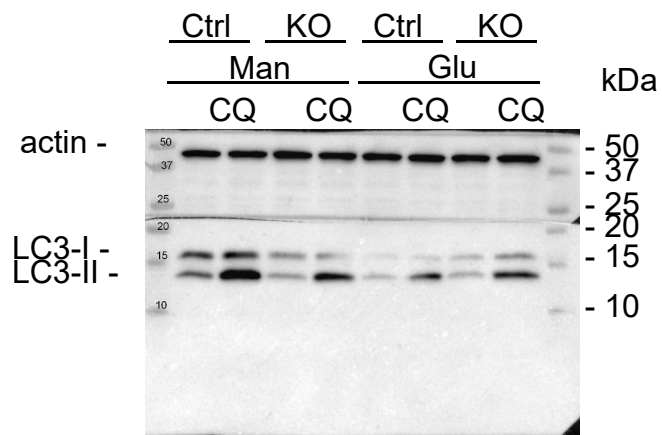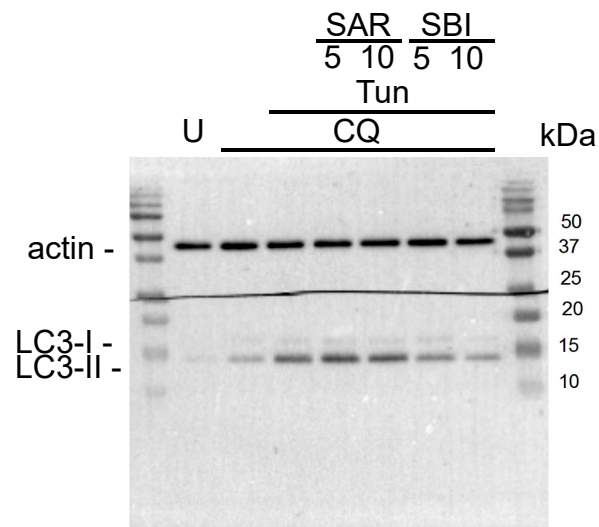

Supplement: S1 Raw images — (PDF) [file pone.0311404.s009.pdf]
